# Changes in 24-Hour Urine Chemistry in Patients with Nephrolithiasis during Weight Loss with Glucagon-Like Peptide 1–Based Therapies

**DOI:** 10.34067/KID.0000000580

**Published:** 2024-09-18

**Authors:** Karen Feghali, Xilong Li, Naim M. Maalouf

**Affiliations:** 1 Department of Internal Medicine, Division of Endocrinology, UT Southwestern Medical Center, Dallas, Texas; 2 Peter O'Donnell Jr. School of Public Health, UT Southwestern Medical Center, Dallas, Texas; 3 Charles and Jane Pak Center for Mineral Metabolism and Clinical Research, UT Southwestern Medical Center, Dallas, Texas

**Keywords:** kidney stones, obesity

## Abstract

**Key Points:**

- In obese kidney stone formers, weight loss with glucagon-like peptide-1 (GLP)-based therapy was associated with a significant decline in 24-hour urine oxalate and sulfate excretion rates.
- Weight loss through GLP-based therapies was associated with nonsignificant changes in urine saturation indices.
- In obese kidney stone formers, GLP-based therapy appears to be a safe option for weight loss on the basis of 24-hour urine studies.

**Background:**

Obesity is an independent risk factor of incident and recurrent nephrolithiasis. The effect of weight loss through glucagon-like peptide 1 (GLP-1) receptor agonists and dual GLP-1/gastric inhibitory polypeptide receptor agonists (GLP-based therapies) on nephrolithiasis is not well understood. This study examined the changes in 24-hour urine chemistry assessing for stone risk during weight loss through GLP-based therapies.

**Methods:**

This retrospective analysis identified adult stone formers followed at our academic institution's weight wellness clinic between September 2015 and August 2023 and included patients with at least two 24-hour urine collections for stone risk assessment. 24-hour urine parameters before and during weight loss in patients on GLP-based therapies were compared.

**Results:**

Forty-four obese patients with nephrolithiasis experienced significant weight reduction (−6.6±7.3 kg, *P* < 0.001) over a median 1.1 years of follow-up with GLP-based therapies. During this period, there was a significant decrease in 24-hour urine oxalate (40±16 to 32±11 mg/d, *P* = 0.002), sulfate (21±10 to 17±9 mmol/d, *P* = 0005), and ammonium (35±22 to 29±15 mEq/d, *P* = 0.01) excretion rates. There were nonsignificant changes in urine calcium, citrate, uric acid, pH, phosphorus, sodium, potassium, magnesium, chloride, creatinine, or total volume. In addition, there was no statistical difference in urine supersaturation indices with respect to calcium oxalate, calcium phosphate, and uric acid.

**Conclusions:**

Our results indicate that weight loss through GLP-based therapies is not associated with prolithogenic changes in 24-hour urine chemistry in patients with nephrolithiasis, unlike what happens with other weight loss modalities.

## Visual Abstract

**Figure:**
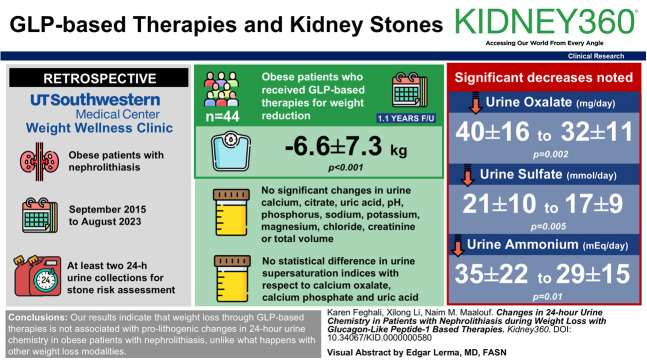

## Introduction

Nephrolithiasis is a major cause of morbidity with a prevalence, reaching 10% in the United States.^1^ The burden of kidney stones is estimated at $10 billion per year, making stone disease one of the most expensive nonmalignant urologic conditions.^2^ The increase in the prevalence of kidney stones has paralleled rampant rates of obesity, which now affects more than 40% of the US population.^3^ In fact, there is ample evidence that obesity is an independent risk factor for the development of kidney stones^1,4–6^ and for stone recurrence after an initial stone.^7^ Obesity-associated nephrolithiasis has been linked to an increased solute load and excess nutrient intake, leading to changes in urine electrolytes, as well as metabolic syndrome and insulin resistance, resulting in decreased urinary pH.^8–10^

Weight loss has been demonstrated to prevent or improve several diseases and conditions associated with obesity, including type 2 diabetes,^11,12^ sleep apnea,^13–15^ osteoarthritis,^16^ and cardiovascular disorders.^17,18^ On the other hand, there are strong data showing an increased incidence of kidney stones during weight loss through malabsorptive bariatric surgery, especially after Roux-en-Y gastric bypass and duodenal switch surgery.^19–21^ This has been attributed primarily to increased gastrointestinal oxalate absorption, leading to greater urinary oxalate excretion,^19–21^ but also to lower citrate excretion and lower urine pH.^22^ In addition, certain pharmacological approaches to weight reduction, such as orlistat and topiramate, may promote kidney stone formation^23^: Orlistat, a lipase inhibitor, leads to fat accumulation in the intestine that binds to calcium, leading to enhanced intestinal oxalate absorption. This in turn leads to increased urinary oxalate excretion, resulting in increased stone risk.^24^ Topiramate, a carbonic anhydrase inhibitor, promotes a decrease in urinary citrate excretion, increase in urinary pH, and hypercalciuria, leading to an increased risk of stone formation.^25^ Weight loss could, therefore, undermine prevention of kidney stones depending on the method with which weight loss is achieved.

Glucagon-like peptide 1 receptor agonists (GLP-1 RAs) and dual GLP-1/gastric inhibitory polypeptide (GIP) receptor agonists (GLP-based therapies) are newer pharmacologic therapies that are used for management of hyperglycemia in type 2 diabetes and are approved for weight loss purposes in the absence of underlying diabetes. They are increasingly prescribed by providers because their effect on weight loss surpasses older weight loss pharmacotherapies.^26,27^ Between 2020 and 2023, the number of adolescents and young adults with GLP-1 RA dispensing increased by nearly 600%.^28^ The effect of weight loss through these recently approved, but increasingly used, drugs on nephrolithiasis risk in patients with known stone disease is not well understood. This is the first study to look at the changes in 24-hour urine chemistry assessing stone risk during weight loss with GLP-based therapies.

## Methods

### Study Design

This retrospective study was conducted to evaluate changes in kidney stone risk in obese patients with a history of nephrolithiasis who were followed at an academic medical center's multidisciplinary weight management program. Patient charts from the electronic medical record were reviewed. The study was approved by the Institutional Review Board of University of Texas Southwestern Medical Center.

### Patient Identification

**Figure 1:**
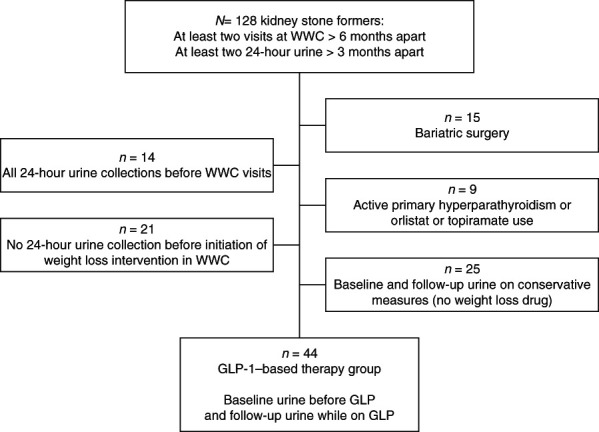
**Flow chart of the cohort.** GLP, glucagon-like peptide; PHPT, primary hyperparathyroidism; WWC, weight wellness clinic.

We first identified patients with history of kidney stones who were 18 years or older and who had at least 2 encounters ≥6 months apart in the weight wellness clinic at the University of Texas Southwestern Medical Center between September 2015 and June 2023 (Figure 1). Among these, we selected patients who had at least two 24-hour urine collections for assessment of stone risk ≥3 months apart. We excluded patients who had a history of bariatric surgery or active and untreated primary hyperparathyroidism and patients on topiramate or orlistat. We further restricted our population by excluding patients who did not have a 24-hour urine collection before initiation of weight loss intervention in the clinic and those with all 24-hour urine collections after initiation of weight loss intervention. Thus, only patients with a single weight loss intervention between the initial and follow-up 24-hour urine collections were included in the study. Next, we identified those patients with at least one 24-hour urine collection before initiation of GLP and at least one 24-hour urine collection while still on GLP. These patients were also counseled on interventions focused on maintaining consistent and balanced meal routines while concentrating on a moderate calorie-restricted diet through portion control, elimination of sugar-containing beverages, and careful meal planning. In addition, aerobic training and resistance exercises to burn calories and increase lean body mass were encouraged.

### Data Extraction

The electronic health records were accessed to extract data on demographic information (age, sex, race, and ethnicity); serial height, weight, and body mass index (BMI); medical and surgical history; stone analysis, medication use; and results of 24-hour urine collections. We extracted baseline weight and BMI immediately before the initiation of the GLP-1–based therapy (closest to the baseline 24-hour urine collection) and furthest follow-up weight and BMI while on GLP-1–based therapy (closest to the follow-up 24-hour urine collection). The GLP-1 medications identified were exenatide, liraglutide, dulaglutide, semaglutide, and tirzepatide. Patients who switched between these drugs were included because they were still on GLP-based therapies. We also extracted data on drugs known to affect urine stone risk profile if patients were on them during baseline and follow-up 24-hour urine collection: allopurinol, sodium bicarbonate, potassium citrate, thiazide and thiazide-like diuretics (indapamide, hydrochlorothiazide, chlorthalidone), and sodium-glucose cotransporter-2 inhibitors.

### Statistical Methods

Weight documented at each clinical encounter was extracted. Results were analyzed comparing the initial 24-hour urine collection and the last follow-up 24-hour urine collection after being on GLP-1–based therapy.

Urine saturation was calculated as supersaturation index (SI) with respect to calcium oxalate, calcium phosphorus (brushite), and uric acid using Joint Expert Speciation System software.^29^

Statistical comparisons were conducted using chi-square test for categorical variables and *t*-test for normally distributed continuous variables or Wilcoxon rank-sum test for non-normal distributed continuous variables. The distribution of the continuous variables was assessed by Shapiro–Wilk normality test and normal probability plots. Continuous variables were presented as mean±SD while categorical variables were expressed as frequency (percentage). Within-group changes (pre- versus post-urine parameters) were compared using paired *t*-test.

All statistical tests were two-sided, and a *P* value of <0.05 was considered statistically significant.

## Results

### Patient Cohort and Characteristics

Table 1 presents the characteristics of the 44 patients included in this analysis. The cohort was predominantly White (68%), non-Hispanic (88%), and female (68%), with an average age of 58 years. The baseline BMI was 36±6 kg/m^2^. Stone analysis was calcium oxalate in 50% of patients, uric acid in 7%, calcium phosphate in 7%, and unavailable in the remaining 36%. Sixty percent of the patients had a history of diabetes.

**Table 1:** Clinical characteristics of the 44 obese stone formers followed longitudinally

Characteristic	Value
Sex, *n* (%) female	30 (68)
Age, yr	58±11
**Race, %**	
Black	18
Other	14
White	68
Ethnicity, % Hispanic	12
**Stone analysis, %**	
Calcium oxalate	50
Uric acid	7
Calcium phosphate	7
Unknown	36
Type 2 diabetes, %	60
Baseline weight, kg	100±22
Follow-up weight, kg	92±20
Change in weight (baseline to follow-up urine), kg	−6.6±7.3 *P* < 0.001
Baseline BMI, kg/m^2^	36±6
Follow-up BMI, kg/m^2^	33±6
Duration of follow-up, yr, median (interquartile range)	1.1 (0.8–2.1)
**GLP-based drugs, %**	
Semaglutide	86
Tirzepatide	48
Lirglutide	21
Dulaglutide	14
**Other medications, %**	
Potassium citrate	45
Sodium bicarbonate	14
Allopurinol	14
Thiazide diuretics	55
SGLT-2i	21

Data shown as mean±SD or percentage. BMI, body mass index; GLP, glucagon-like peptide; SGLT-2i, sodium-glucose cotransporter-2 inhibitor.

The GLP-based therapies used during the weight loss intervention included semaglutide (86%), tirzepatide (48%), liraglutide (21%), and dulaglutide (14%), with patients switching between these agents on the basis of insurance coverage and/or medication availability.

During a median follow-up duration of 1.1 years, average weight loss was −6.6±7.3 kg (*P* < 0.001).

### Changes in 24-Hour Urine Parameters with Weight Loss

Twenty four-hour urine parameter results before and after weight loss intervention are presented in Table 2.

**Table 2:** Longitudinal changes in 24-hour urine chemistry associated with weight loss with GLP-1–based therapies

24-h Urine Parameter	Pre-GLP	During GLP	*P* Value
Oxalate, mg/d	40±16	32±11	0.002
Sulfate, mmol/d	21±10	17±9	0.005
Ammonium, mEq/d	35±22	29±15	0.014
Uric acid, mg/d	632±290	551±225	0.052
Creatinine, mg/d	1485±479	1357±469	0.063
Phosphorus, mg/d	869±378	745±338	0.11
Calcium, mg/d	250±160	217±133	0.13
pH	6.14±0.66	6.22±0.63	0.64
Total volume, L/d	2.15±0.93	2.03±0.89	0.14
Citrate, mg/d	726±490	651±362	0.23
Sodium, mmol/d	189±99	237±59	0.19
Chloride, mmol/d	170±97	151±85	0.21
Potassium, mmol/d	66±33	109±44	0.45
Magnesium, mg/d	112±57	116±60	0.72

GLP, glucagon-like peptide.

**Figure 2:**
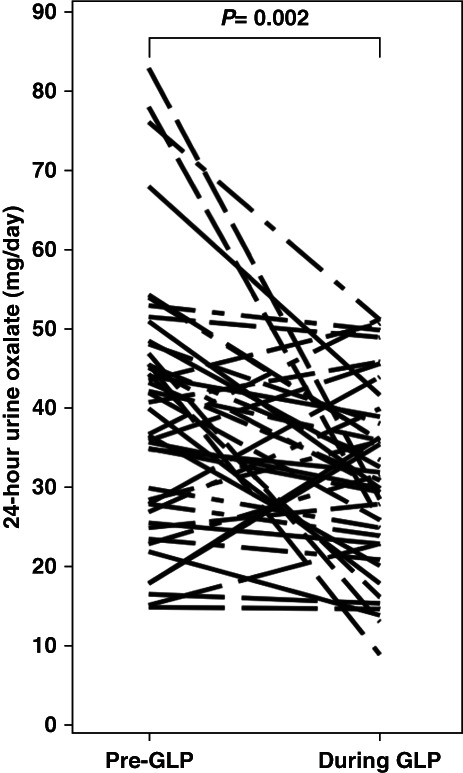
Change in 24-hour urine oxalate excretion during weight loss with GLP-1–based therapies.

There was a significant decrease in 24-hour urine oxalate (40±16 to 32±11 mg/d, Figure 2), sulfate (21±10 to 17±9 mmol/d), and ammonium (35±22 to 29±15 mEq/d) concentrations. There was no significant change in 24-hour urine calcium, citrate, uric acid, pH, phosphorus, sodium, potassium, magnesium, chloride, creatinine, or total volume.

**Figure 3:**
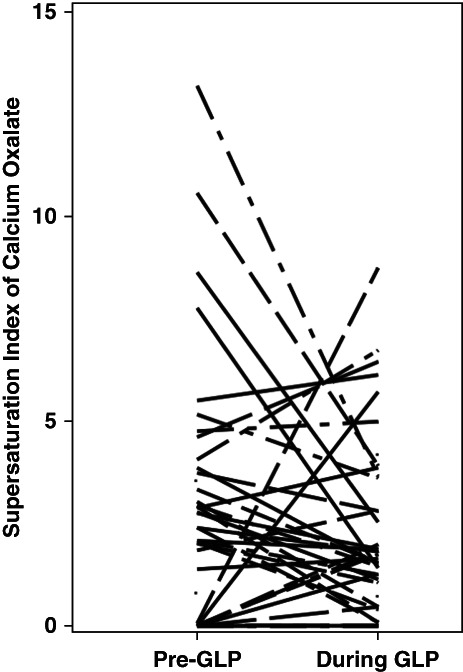
**Change in SI with respect to calcium oxalate during weight loss with GLP-1–based therapies.** SI, supersaturation index.

There was no statistical difference in urine SI with respect to calcium oxalate (2.76±3.01 to 2.24±2.17) (Figure 3). Moreover, there was no significant change in urine SI with respect to calcium phosphate (0.74±0.99 to 0.63±0.58) or uric acid (1.09±1.07 to 1.00±1.12) in the GLP group (Table 3).

**Table 3:** Urinary saturation indices before and after weight loss treatment

Stone Risk	Pre-GLP	During GLP	*P* Value
SI calcium oxalate	2.76±3.01	2.24±2.17	0.27
SI calcium phosphate	0.74±0.99	0.63±0.58	0.43
SI uric acid	1.09±1.07	1.00±1.12	0.78

GLP, glucagon-like peptide; SI, supersaturation index.

## Discussion

An increasing population of obese patients with nephrolithiasis is seeking weight loss through various surgical, dietary, and pharmacological therapies. This is the first study to examine the effect of the increasingly prescribed GLP-based therapies on 24-hour urine chemistries assessing stone formation risk. Our results show that weight loss though GLP-based therapies is associated with a significant reduction in 24-hour urine oxalate, urine sulfate, and urine ammonium concentrations, along with nonsignificant changes in other 24-hour urine parameters relevant to stone formation. Overall, there were no significant changes in urinary saturation with respect to calcium oxalate, calcium phosphate, or uric acid. Our results suggest that GLP-based therapies can be a safe approach for weight loss in patients with history of nephrolithiasis, although longer term studies following a larger population of obese stone formers will be needed to definitively confirm the effect of GLP-based therapy on stone incidence and recurrence.

Whether our findings are related to change in dietary intake, to weight loss by itself, or to direct effects through GLP-1 receptors on kidneys cannot be determined from our study. The incretin hormones GLP and GIP are secreted from the gut in response to nutrient ingestion and stimulate insulin secretion by binding to specific receptors in pancreatic beta cells.^30^ Besides their antihyperglycemic action, they play a key role in reducing appetite, food intake, and ultimately body weight.^31^ As such, GLP-1 RAs and dual GLP/GIP-RAs have emerged as essential drugs for the treatment of patients with type 2 diabetes and, more recently, for the management of weight loss in the absence of diabetes.

Interestingly, the role of GLP-1 is not limited to the pancreas because GLP receptors are present on various organs, including kidneys.^32^ The presence of these receptors in renal tissue suggests that GLP-1 has specific renal effects with growing evidence supporting a role for GLP-1 in modulating kidney function.^32,33^ The limited studies that evaluated the renal effect of GLP-1 RAs have mostly focused on their natriuretic properties. Current evidence suggests that activation of the GLP-1 RA in vascular smooth muscle of the renal vasculature leads to an increase in urinary flow and sodium excretion.^34–36^ Fewer publications have examined the effect of GLP-1 RAs on other urine electrolytes, and the results were inconsistent. In one study of healthy obese male patients, a 3-hour intravenous infusion of GLP-1 caused significant increases in sodium, chloride, and calcium excretion, accompanied by a significant reduction in hydrogen excretion.^37^ Similar increases in urine sodium and urine pH were reported with an acute infusion of the GLP-RA exenatide.^38^ Other acute studies in healthy nonobese male individuals did not identify a change in urinary hydrogen excretion or urinary pH.^35,36^ Overall, these studies did not show a significant change in urine potassium concentration.^35–38^ Regarding urine potassium, our results, like available data, showed no significant change with GLP therapy. On the other hand, contrary to existing studies, our results show nonsignificant changes in urine sodium, chloride, calcium, or pH. The differences between our results and prior publications are likely due to several factors. In the previously published studies, only the short-term effects of GLP-1 RA administration on urine parameters were assessed. In addition, these studies only included men, with no mention of kidney stone status, nor weight changes. On the other hand, our study population consisted exclusively of kidney stone formers, including both men and women, who were followed for a longer duration of time (approximately 1 years). Therefore, most of our patients experienced significant weight loss, with likely changes in food intake, which in turn alters urine chemistry. Moreover, a large proportion of our patients were on newer GLP-based therapies, such as semaglutide and tirzepatide.

To date, this study is the first to evaluate the effect of GLP-based therapies on urine oxalate, urine citrate, or urine uric acid. The pathophysiology underlying the significant decrease in urine oxalate concentration seen in the GLP group is complex. Urine oxalate excretion is positively correlated with weight and BMI, and urinary oxalate is significantly higher in obese compared with lean adult calcium oxalate stone formers.^23,39^ This urinary oxalate pool is derived from both dietary oxalate intake and endogenous oxalate synthesis in the liver.^40^ In addition, metabolic dysfunction-associated steatohepatitis (MASH, previously known as nonalcoholic steatohepatitis) has been identified as a risk factor of hyperoxaluria through impaired detoxification of the oxalate precursor glyoxylate.^41^ The significant reduction in urinary oxalate excretion after weight loss through GLP-based therapies could possibly be attributed to decreased intake of oxalate and oxalate precursors or to reduced hepatic oxalate synthesis. GLP-based therapies have been extensively studied in fatty liver disease, with liraglutide improving liver enzymes, hepatic steatosis,^42,43^ and semaglutide contributing to resolution of MASH.^44^ The potential mechanism of action of GLP-1 RAes in MASH may be related to the effects on weight and insulin resistance, as well as reductions in metabolic dysfunction, lipotoxic effects, and inflammation.^45,46^ Given the extensive effect of GLP-based therapies on hepatic metabolism and the proven benefits on fatty liver, it is possible that these agents reduce endogenous hepatic oxalate synthesis in addition to their effect on dietary oxalate intake.

The significant decrease in 24-hour urine sulfate, a marker of dietary animal protein intake, observed in our study could be attributed to the known effects of reduced appetite and nausea observed with these agents and, ultimately, from decreased protein consumption. The decrease in 24-hour urine ammonium, a major urinary buffer, likely parallels the change in urine sulfate excretion and likely represents decreased exposure to the acid load imparted by animal proteins.^47^ In addition to the desired decrease in body weight with GLP-based therapies, these agents also result in decreased lean mass, including muscle mass.^48,49^ In our study, there was a trend for a decrease in urine creatinine excretion observed after weight loss (*P* = 0.06), which is likely explained by the decrease in muscle mass. Interestingly, 24-hour urine total volume did not change significantly. Although GLP-based therapies reduce appetite and occasionally cause nausea, they do not seem to significantly alter fluid consumption in our study.

Our study has several clinical implications. Available studies clearly show that weight loss after bariatric surgery^19–21^ or older weight loss pharmacotherapies, such as orlistat and topiramate,^23–25^ increase the risk of incident kidney stone formation. The lack of significant change in urinary saturation with respect to calcium oxalate, calcium phosphate, and uric acid seen in our study points to the safety of GLP-based therapy as an option for weight loss for obese kidney stone formers.

There are several limitations to our study. The retrospective, nonrandomized design prevents us from separating the effect of weight loss *per se* from changes in dietary intake (which by itself could affect 24-hour urine parameters). Although some studies have assessed 24-hour urine parameters collected on a fixed metabolic diet, diet in our study was not controlled. However, it is impractical to keep patients on the same diet longitudinally, especially because GLP-based therapies alter appetite and food intake. Second, our patient population was relatively small. Nevertheless, most stone formers do not routinely undergo 24-hour urine collections,^50^ and an even smaller population undergoes a repeat urine collection.^51^ Third, our study exclusively examined the effect of GLP-based therapy on 24-hour urine chemistry and did not assess for new stone events, an outcome that should be examined in the context of larger longitudinal studies.

In summary, our urine results indicate that weight loss through GLP-based therapies is not associated with prolithogenic changes in 24-hour urine chemistry in patients with nephrolithiasis, unlike what happens with alternative weight loss modalities (such as malabsorptive bariatric surgery and pharmacological therapy with orlistat or topiramate). Additional larger prospective studies are needed to further assess the mechanism of action of these drugs on the kidney and, hence, their effect on kidney stones.

## Data Availability

All data are included in the manuscript and/or supporting information.
